# Germination Data Analysis by Time-to-Event Approaches

**DOI:** 10.3390/plants9050617

**Published:** 2020-05-12

**Authors:** Alessandro Romano, Piergiorgio Stevanato

**Affiliations:** 1 Plant Protection and Certification Centre, Council for Agricultural Research and Economics, 36045 Lonigo, Italy; 2 Department of Agronomy, Food, Natural Resources, Animals and Environment, University of Padova, 35020 Legnaro, Italy; stevanato@unipd.it

**Keywords:** germination, sugar beet, water stress, survival analysis, accelerated failure time model, Cox’s proportional hazard model, Kaplan Meier estimator

## Abstract

Germination data are analyzed by several methods, which can be mainly classified as germination indexes and traditional regression techniques to fit non-linear parametric functions to the temporal sequence of cumulative germination. However, due to the nature of germination data, often different from other biological data, the abovementioned methods may present some limits, especially when ungerminated seeds are present at the end of an experiment. A class of methods that could allow addressing these issues is represented by the so-called “time-to-event analysis”, better known in other scientific fields as “survival analysis” or “reliability analysis”. There is relatively little literature about the application of these methods to germination data, and some reviews dealt only with parts of the possible approaches such as either non-parametric and semi-parametric or parametric ones. The present study aims to give a contribution to the knowledge about the reliability of these methods by assessing all the main approaches to the same germination data provided by sugar beet (*Beta vulgaris* L.) seeds cohorts. The results obtained confirmed that although the different approaches present advantages and disadvantages, they could generally represent a valuable tool to analyze germination data providing parameters whose usefulness depends on the purpose of the research.

## 1. Introduction

Germination is one of the most crucial physiological processes that allows plants to establish in a particular environment. Germination experiments are carried out in several fields of biological sciences [1]. Many of these experiments involve determining the germination percentage of seeds germinated after specified time intervals by repeated observations and/or the calculation of the germination rate [2].

Several methods of analyzing the resulting data have been reviewed, mainly germination indexes [3] and classical regression techniques to fit non-linear parametric functions to the temporal sequence of cumulative germination [4,5]. There is an exhaustive literature about all these techniques and many experiments have been performed on several species.

Another class of methods to analyze germination data, still not largely used, is given by time-to-event analysis. Such approaches are often referred to as survival or reliability analysis ([6], pp. 2–5) and are well known and widespread methods in other scientific fields, as applications are often concerned with, among others, the reliability in engineering and devices [7] the failure time of machine components in industrial processes [8], the time to death or recovery of patients in clinical trials [9,10], psychological experimentation [11]. Within biological fields, works about plant pathology [12], and entomology [13,14] were done as well.

The method describes a binary response variable, where data are collected as cumulative counts over time, and consists of modelling the time to response for each individual in the sample. With regard to germination studies, data can be collected as the germination time of each seed, as the number of seeds germinating in each time interval or the cumulative number of seeds which have germinated by the observation time. Time-to-event analysis can be approached by three different ways: non-parametric, semi-parametric and fully parametric.

Non-parametric methods make no assumptions about an underlying probability distribution. That is how the event of germination changes over time, based on the probability of seed development. Using non-parametric methods, we estimate and plot the survival distribution or survival curve.

It is possible to compare two or more survival distributions. To this regard, the log-rank test [15], also known as the Mantel log-rank test, the Cox Mantel log-rank test and the Mantel Haenszel test and its versions, are the most widely used methods.

Survival analysis methods can also be extended to assess several risk factors or exposures simultaneously similar to multiple linear and multiple logistic regression analysis. The Cox’s Proportional Hazard model is the most general of the regression models because it is not based on any assumptions concerning the nature of shape of the underlying survival distribution. In this model, the response variable is the ‘hazard’. The model assumes that the underlying hazard rate, rather than survival time, is a function of the independent variables or covariates [16].

In fully parametric models, a specific probability distribution of the baseline hazard/survival is assumed, according to a defined probability distribution. Parametric models can be expressed in: (1) proportional hazard form, where a one-unit change in an explanatory variable causes proportional changes in hazard; and (2) Accelerated Failure Time (AFT) form, where a one unit change in an explanatory variable causes a proportional change in survival time. AFT model is calculated as a function of the number of seeds that have germinated at each time interval prior to that point in time, and the number of seeds that could have germinated at the end of the preceding interval. The AFT approach allows considerations for a range of statistical distribution functions for the times to germination of seeds in a seed-lot and compares the responses for different seed-lots through estimation of an ‘acceleration factor’ that summarizes the relative germination rates of the seed-lots but assumes a common shape of response. McNair et al. [17] reviewed exhaustively non-parametric and semi-parametric models applied to germination data, whereas Onofri et al. [18] considered a range of AFT models to describe germination responses for different weed species. Other authors applied these methods to their research, more precisely non-parametric and semi-parametric models [19,20,21,22,23,24,25,26,27] and AFT model [28,29].

However, to our knowledge, no works have examined the application of non-parametric, semi-parametric and fully parametric models to the same data comparing the biological meaning of the estimated parameters, therefore the aim of the present work is to contribute to the knowledge of the effectiveness of time-to-events methods by analyzing germination data of sugar beet seeds, using two categorical covariates, osmotic stress and genotype, and comparing the three most common approaches to verify the reliability of the results and do the groundwork for more articulated future experimentations.

## 2. Results and Discussion

### 2.1. Kaplan-Meier Estimator

**Figure 1:**
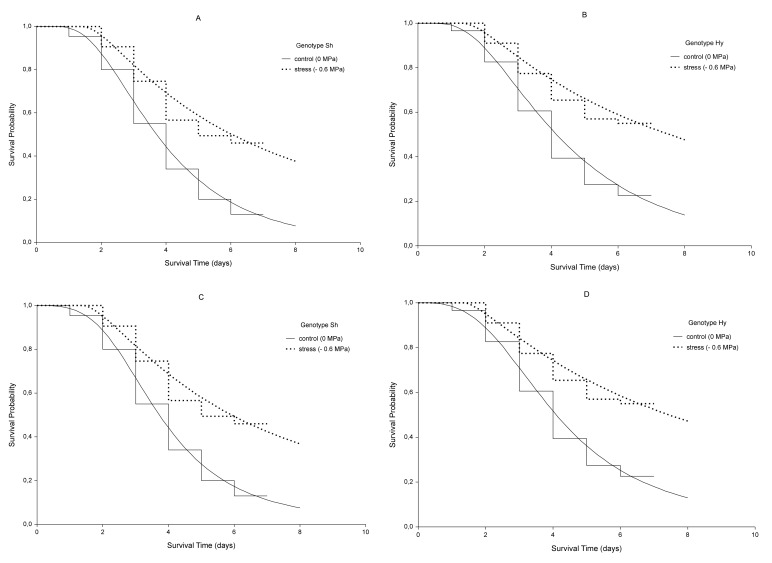
Kaplan-Meier estimates of survival functions (step curves) and AFT log-normal (**A**,**B**) and log-logistic (**C**,**D**) model curves (continuous curves) for genotypes Sh and Hy. Dotted lines indicate the stress condition (*ψ* = −0.6 MPa), continuous lines indicate the control (*ψ* =0 MPa).

Kaplan-Meier (KM) step curves are shown in Figure 1. The non-continuous nature of KM curves highlights the fact that they are not smooth functions, but rather stepwise estimates. The value of *S(t)* is constant between times of germination events, and the estimated probability changes value only at the time of each event. The vertical distances between horizontals represent the change in cumulative probability of not germinating as the curve advances. The steepness of the curve is determined by how long the survival lasts and is represented by the length of horizontal lines.

Whereas the cumulative probability, seen on the Y-axis of the curve, represents the probability at the beginning and throughout the interval, the interval survival rate represents the probability of not germinating after the interval and at the beginning of the next.

For the control sample of genotype Sh, the cumulative incidence or cumulative germination probability, expressed as 1–*S_t_*, at the 4th day is 1–0.55 (45%); conversely, in presence of stress, the cumulative incidence at the same time is 1–0.745 (25.5%). Considering the same days, for genotype Hy, the cumulative germination probability is 1–0.605 (39.5%, Control) and 1–0.775 (22.5%, −0.6 MPa).

Reasoning in terms of interval survival rate, the first interval starts at the baseline time (*t* = 0) and ends just before the first germination event. The survival rate for this interval is 1, which means no seeds have still germinated. For example, at serial time 1 day, for control Sh, a few seeds germinated, and the chance of not germinating past 1 day, drops to 0.955 in the second interval, to 0.837 in the third interval and so on.

The rates are given by the ratio between the number of not germinated seeds (“at risk”) in an interval and that of the previous one. Cumulative probabilities for an interval are calculated by multiplying the interval survival rates up to that interval. Then, the cumulative aforementioned probabilities are the results of these multiplications. It is also possible to reason in terms of non-parametric hazard rates and cumulative hazard. The first represents the instantaneous germination rate at any point in time given by the ratios between germinated seeds and latent seeds, the cumulative hazard function is the integral of the germination rates from time 0 to 7th day, which represents the accumulation of the hazard over time.

The intersection between a horizontal line associated with the probability of survival equal to 0.5 and the survival curve in Figure 1 allows us finding the median germination time. In both genotypes, it was about 4 days for control and 6 days for stressed seeds. However, because the genotype Hy did not reach a 50% final germination under stress, its median germination time is not clearly defined. The median is a better measure of centrality than the mean because of the skewness of survival times. Furthermore, we do not know if not germinate seeds would have germinated later in the present study.

Comparisons of survival functions were done between genotypes, both with and without PEG treatment and within each genotype between stress and no-stress condition. Table 1 shows the results of the tests applied to germination data. The *p*-values were calculated to test the significance of differences between groups for *α* = 0.05. As it is possible to notice, the values of *z*, χ^2^ and the level of significance *p* are different in accordance with the kind of test applied.

The most evident differences exist between control and stressed seeds for both genotypes, where all *p*-values were <0.0001 irrespective of the weight given by the different tests to specific regions of the whole period. Conversely, only the M-H log-rank and the F-H test showed significant differences between the non-stressed seeds of the two genotypes letting us infer that the osmotic stress affected the germination irrespective of the germination capability in standard conditions (0 MPa).

**Figure 2:**
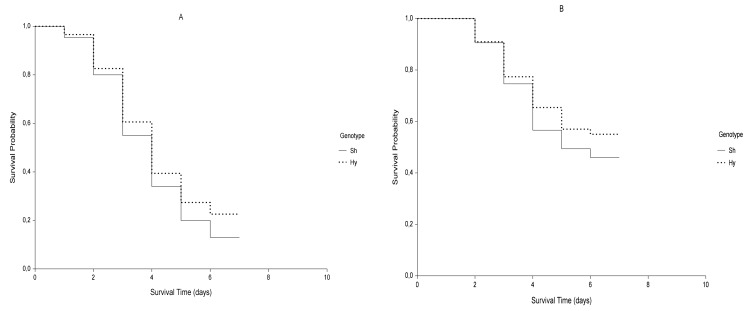
Kaplan-Meier estimates of survival functions of control (**A**; *ψ* = 0 MPa) and stressed (**B**; *ψ* = −0.6 MPa) samples for genotypes Sh (continuous curve) and Hy (dotted curve).

It could be interesting to note that for germination data, when differences in survival curves do not appear evident (Figure 2) the M-H log-rank (*q* = *p* = 0) and the F-H test (*q* = 0; *p* = 1) were the only ones that detected significant differences between the genotypes, the first placing the same weight on all germination times throughout the whole time period and the second one placing a little more weight at the end of the germination period. Specifically, F-H test was consistent with the trend of germination, expressed in terms of hazard rates of the two genotypes. Hy slowed down the germination rate later compared to Sh, whereas the differences at the beginning and in the middle were slighter.

### 2.2. Cox’s Proportional Hazard

The assumptions of Cox’s Proportional Hazard model were fulfilled. Plotting preventively the survival function versus the survival time and the log(−log(survival)) versus the log of survival time, we obtained parallel curves and parallel lines, respectively for every combination genotype/treatment.

Table 2 shows the parameters calculated by the application of Cox’s PH model considering the Sh control as a reference group. The negative sign of the regression coefficients *β*_i_, −0.23 for genotype and −0.87 for treatment, indicates that the genotype Hy has a lower probability of germination than Sh and the stress condition leads to the same result if compared with no stressed seeds.

The exponentiated coefficients, exp (*b*_i_) represent the hazard ratios and give the effect size of covariates. They can be considered as the predicted change in the hazard for a unit increase in the predictor.

The value of hazard ratio equal to 0.79 (<1) indicates that the effect of the variable “genotype”, will decrease the hazard function throughout the observation period by 21%, holding the “treatment” covariate constant. The reverse 1/exp (*β*_i_) equal to 1.26, considering the codes of genotype Sh = 0 and that of genotype Hy = 1, indicates that the probability of experiencing germination increases by a factor 1.26 when genotype is Sh compared with genotype Hy. The *p*-value for genotype is 0.0072, indicating a strong relationship between the genotype covariate and the decreased chance of germination.

Equally, with respect to the treatment variable, holding the genotype covariate constant, a hazard ratio equal to 0.42 suggests that the stress treatment decreases the hazard function by 58%. The reverse 1/exp (*β*_i_), considering the codes for stress = 0 and for no stress = 1, indicates that the probability of germinating increases by a factor 2.38 for no stressed seeds compared to the stressed ones. The *p*-value for treatment is lower than 0.0001, indicating a very strong relationship between the stress treatment and the decreased probability of germination. Thus, both predictors, genotype and treatment, turned out to be “protective” from germination.

The 95% confidence intervals (CI) for the hazard ratios do not overlap the values of 1, indicating a strong relationship between both predictors, genotype and PEG treatment, and decreased germination (Table 2).

### 2.3. Accelerated Failure Time Model

**Figure 3:**
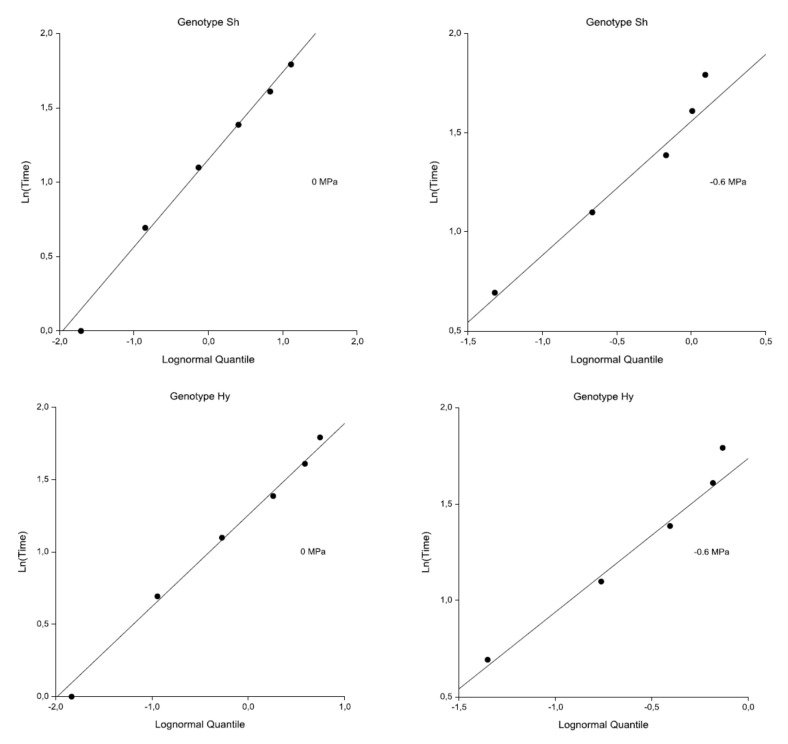
Log-normal distribution probability plots for Sh and Hy genotypes. On the horizontal axis, the expected quantile of the theoretical distribution, Φ^−1^(1 − e^−H(t)^), where H (t) is the cumulative hazard rate and Φ is the standard normal distribution function; on the vertical axis, the natural logarithm of the time value. For tied data, only one point is shown for each set of ties.

Figure 3 shows the probability plots of the log-normal distribution for both genotypes and treatments. The straight lines obtained suggest an adequacy of the model for our data. With respect to the tied data, only one point is shown for each set of ties. The intercept and slope values of the lines in Figure 3 are graphical estimates of the mean and variance of the log-normal distribution. The values are close to those calculated by the maximum likelihood estimates (MLEs) reported in Table 3. The probability plots of log-logistic distribution for both genotypes and treatments had a similar linearity.

The application of the Akaike Information Criterion (AIC) confirmed that the best distributions for our experiment were the log-normal and the log-logistic.

The log-normal distribution for hazard function is not always considered appropriate in other fields since its pattern considers a starting value equal to 0, increases up to a maximum with increasing time and eventually decreases to zero as *t* becomes large, and in many analyses the hazard function will generally not approach zero at large time, since all the objects will eventually fail [30]. However, this pattern seems to fit the ordinary trend of the germination process rendering this model suitable for our results.

The survivorship estimates of the log-normal for the stress and no-stress treatment are shown in Figure 1 (continuous curves). The model roughly fits the survival data as represented by step-curves.

Table 4 shows maximum likelihood estimates (MLE) of the parameters calculated through the application of parametric model expressed as Accelerated Failure Time (AFT) regression.

Looking at the log-normal model table, the intercept value for the reference seed lot (genotype Sh and no-stress treatment) is represented by *α*_0_ and assumes the value of 1.32. Stress treatment (0.45) and genotype Hy (0.11) effects represent the increases in log-time, the time past which seeds do not germinate, due to PEG application and the “genotype factor”, respectively. The T50 for reference seed lot, calculated as exp (*α*_0_), was 3.7 days, whereas considering the “treatment” effect exp (*α*_0_ + *α*_1_), and the “genotype” effect exp (*α*_0_ + *α*_2_), T50 was about 5.9 days and 4.2 respectively.

In terms of time ratio, interpreted as the estimated ratios of the expected survival times for two groups, it can be observed that the application of stress treatment slows down germination by 57%, whereas the genotype effect is lower (12%).

In the AFT framework, a time ratio γ > 1 for the covariate implies that this covariate prolongs the time of germination, while a time ratio γ < 1 indicates that an earlier germination event is more probable. Similar results were obtained by the application of log-logistic model (Table 4).

Looking at Figure 1, we can observe that the effect of the only stress covariate is a stretch of the survival curve along the time axis by a constant relative amount γ. Being γ > 1, the time of germination increases. The survival probabilities, *S*(*t*), for control sample and stressed seeds are *S*_0_(*t*) and *S*_0_(γ*t*), respectively. The proportion of seeds which are event-free, i.e., not germinated, in stressed sample at any time point *t*_1_ is the same as the proportion of those which do not germinate in the control sample at a time *t*_2_ = γ*t*_1_.

### 2.4. General Considerations

In the present work, Kaplan-Meier (KM) step curves belonging to the two genotypes for each water potential do not cross each other, although a slight partial overlap can be noticed at the beginning, and this could lower the statistical power of this approach compared to the parametric model. Furthermore, being a univariate model, it considers only a factor at a time, genotype or stress level, whereas germination event is a result of several factors that interact at the same time.

The application of semi-parametric and parametric models allows to overcome these limits. The Cox’s PH model provides useful information by considering the two covariates, genotype and stress level, affecting germination simultaneously. In the Cox’s PH model, the covariates are multiplicatively related to the hazard, not to the actual survival time. However, proportional hazard model does not permit a useful parametric distinction between the effects of factors on germination time and the effects of factors on the limiting survival probability of the event.

Whereas the Cox’s model expresses the multiplicative effect of explanatory variables on the hazard (hazard scale), the AFT model expresses the same effect on survival time (time scale). This feature allows for an easier interpretation of the results because parameters measure the strength and the effect of the correspondent covariate on median survival time. By the accelerated failure time application, the effect of covariates on survival is described in absolute terms (e.g., numbers of days) rather than relative terms such as hazard ratio. Furthermore, time ratios could represent useful indexes that outline the effects of explanatory variables on germination and more interpretable than a ratio of two hazards.

Looking at the results reported on the tables, information from the AFT model looks easier to interpret, more relevant, and provide a more appropriate description of germination data. In the present work, we chose an appropriate parametric form that, usually, makes the AFT model more powerful than the semi-parametric one [31].

However, as observed by Onofri et al. [18], AFT models assume that every individual of the cohort under investigation will experience the event sooner or later. It means that the curve of cumulative proportion of germinated seeds should approach an asymptote with time tending to infinity. This is often unrealistic for seeds, considering that many of them could have lost the ability to germinate owing to unfavorable environmental conditions. Although some studies were done to handle these limits [32], in this case other methods could be used in place of AFT models.

## 3. Material and Methods

### 3.1. Germination Experiment

The experiment was carried out on sugar beet seeds of two genotypes: the commercial variety “Shannon”, provided by Lion Seeds Ltd. (Maldon, UK), and a hybrid derived from a breeding program at DAFNAE, University of Padova (Padova, Italy). In the present work, the two genotypes will be named as Sh and Hy respectively. Seeds were scarified with 3% (*v/v*) hydrogen peroxide, continuously stirred for about 14 h and then washed thoroughly with deionized water. Then, 5 replicates consisting of 40 seeds for each genotype and treatment were placed in Petri dishes (Ø = 9 cm) containing two filter paper disks moistened with deionized water (control) and a solution of polyethylene glycol (PEG) 8000 (cat. P2139, Sigma Aldrich, St. Louis, MO, USA), to reach an osmotic potential of −0.6 MPa, calculated using the equation by Michel [33].

Petri dishes were sealed with Parafilm^®^ and kept for 7 days in the dark at a temperature of 25 °C and 70% relative humidity. In order to keep the variable “treatment” constant over time, seeds were transferred in Petri dishes with fresh solutions every 2 days. The number of germinated seeds was recorded daily until the 7th day, and after the counting, seeds were removed from the Petri dishes. Seeds showing at least a 2 mm-long radicle were considered as successfully germinated. On the 7th day the number of not geminated seeds was recorded as well.

### 3.2. Data Collection and Statistical Analysis

In our experiment we adopted the “continuous observations” scheme since we assumed that germination times were known exactly for each seed according to McNair et al. [17]. Furthermore, we did not have “lost” seeds, but the germination time for the seeds that did not germinate was considered as “right-censored”.

The treatment variable was considered as categorical covariate as well as the genotype variable. All seeds were coded as “1” for those germinated, and “0” for those not germinated by the end of the experiment (right-censored observations). The same codes were used to discriminate between genotype Sh (code 0) and genotype Hy (code 1) and between control (code 0) and stress treatment (code 1) for semi-parametric and fully parametric methods. Germination data were submitted to a survival analysis by the statistical software NCSS 12 Data Analysis (NCSS, LLC, Kaysville, UT, USA).

### 3.3. Non-Parametric Approach

Germination data were described and modelled in terms of two related probabilities: survival and hazard functions, both dependent on time. The survival function *S*(*t*) is defined as the probability of surviving at least to time *t*. The hazard function *h*(*t*) is the conditional probability that the investigated event will happen at the same time *t* having survived to that time ([34], p. 92). The hazard function is mathematically related to survival function: the faster the survival function decreases over time, the higher the hazard.

Translating these concepts in terms of germination, survival function *S*(*t*) is considered as the likelihood that a seed will not germinate during its follow-up time elapsing from the time origin, the time at which seeds were put in Petri dishes and incubated in a climatic chamber, to the specified future time *t*, the time at which germination event will occur or observations will be finished. The hazard function *h*(*t*) is the conditional probability that a seed under observation not still germinated at time *t* will germinate shortly after that time. It can also be considered as the instantaneous germination occurrence rate for a single seed, not previously germinated, that has already arrived at the time *t*. To sum up, the hazard relates to the incident event rate, while survival reflects the cumulative non-occurrence [35]. More correctly, the value of the hazard function is not a probability, but it is an indicator of the chance of experiencing the germination event by a seed and it has units of 1/*t*. The higher the value of *h*(*t*), the higher the “risk” of germination [36]. The hazard rate was estimated by statistical software using kernel smoothing of the Nelson-Aalen estimator as given in Klein and Moeschberger ([34] (pp. 166–168).

The survival probability was estimated non-parametrically from observed germination times, both censored and uncensored, using the Kaplan-Meier (KM), or product-limit, estimator [37].

The probability of not germinating at time *t_j_*, *S*(*t_j_*), was calculated from *S*(*t_j_*_-1_), that is the probability of not germinating at *t_j_*_-1_ according to the following formula:

*S*(*t_j_*) = *S*(*t_j_*_−1_) (1 − *d_j_*/*n_j_*) (1)

where *n_j_* is the number of seeds not still germinated but potentially susceptible to germination just before *t_j_*, and *d_j_* is the number of germination events at *t_j_*. At the beginning of the study, baseline time (*t*_0_) = 0 and *S*(0) = 1.

Plotting the KM survival probability against time, survival curves were built, and the median survival time was calculated. Linear pointwise confidence intervals for the survival probability at a specific time point *t*_0_ of *S*(*t_0_*) were also calculated by the Greenwood’s formula.

Since germination of every seed is assumed to occur independently of one another, the probabilities of germinating from one interval to the next was multiplied together to obtain the cumulative hazard function, *H*(*t*), by the Nelson-Aalen estimator, given as *H(t)*= −ln[*S(t)*].

In order to test the null hypothesis of no differences between the survival curves of the two tested sugar beet genotypes and between the control and the osmotically stressed sample within each genotype, a set of log-rank tests was performed [38,39,40,41,42,43]. The log-rank test is based on the following statistics:

*Z_j_*(*τ*) = ∑*^D^*_*i*=1_*W_j_*(*t_i_*) (*O_ij_* − *E_ij_*) (2)

*t_1_, t_2_…t_D_* are the distinct germination times in the pooled sample; *O_ij_* is the observed number of events at time *t_i_* in sample *j*; *E_ij_* is the corresponding expected number of events; *j* are the k groups which are compared, in our case *j* = 2; *W_j_*(*t_i_*) is a positive weight function, whose value gives a different importance on successive event times; τ is the largest time at which there is at least one seed susceptible to germination in every group.

Being only two groups compared, the log-rank test verifies the null hypothesis if the ratio of the hazard rates in the two groups is equal to 1. The hazard ratio (HR) is a measure of the relative survival experience in the two groups. If z represents the vector of *k*-1 statistics and Σ represent the variance-covariance matrix, the test statistics is given by *Q* = *Z*(*τ*)Σ^−^^1^*Z*(*τ*)^t^ [34] (p. 219).

The Mantel-Haenszel log-rank statistics is approximately distributed as a chi-squared test statistic with a k-1 degree of freedom (df = 1 in our case). The test is based on the size of *Q*: if enough large, the null hypothesis that there is no difference between observed and expected values could be rejected ([34], p. 217).

In the present work, in addition to the Mantel-Haenszel (M-H) log-rank test, other weighted two-sample tests for survival data were performed: Gehan’s generalised Wilcoxon test (also known as Breslow’s test); Peto-Peto’s Wilcoxon test, Tarone-Ware test, modified Peto-Peto test and Fleming-Harrington (F-H) test. All these tests differ from each other in their weight function *W*(*t_i_*), that is they emphasize certain times more than others. The choice of the weight function in F-H test was made before evaluating the data and based on expectations for the outcome [44,45].

### 3.4. Semi-Parametric Approach

In order to assess the effects of two risk factors (stress and genotype) on germination function at the same time, we approached survival analysis in a multivariate way using the Cox’s Proportional Hazards regression (PH model), which relates several risk factors or exposures, considered simultaneously, to survival time [46].

Before applying the model, the assessment of the proportional hazards assumptions were made by a graphical method that works best for time fixed covariates with few levels, plotting the survival function versus the survival time and, similarly, the log(−log(survival)) versus log of survival time, in order to make sure that the first graph showed parallel curves, whereas the second one showed parallel lines if the predictor was proportional [47].

PH model calculates the hazard rate that is the chance of germination (i.e., the probability of “suffering” the event of interest). The Cox’s PH regression model can be written as follows [16]:

*h*(*t*) = *h_0_*(*t*) exp (*β*_1_*z*_1_ + *β*_2_*z*_2_ +…+ *β_m_z_m_*) (3)

where *h*(*t*) is the expected hazard at time *t*, given the values of the *m* covariates for the respective case (*z*_1_ and *z*_2_,…, *z*_m_) and the respective survival time (*t*); *z_1_*, *z_2_*, *z_m_* are the predictors, or explanatory variables; *h_0_(t)* is the baseline hazard and represents the hazard for the respective individual when all independent explanatory variables are equal to zero; *β*_1_, *β*_2_,…, *β*_m_ are regression coefficients.

Notice that the predicted hazard *h(t)*, in the next instant, is the product of the baseline hazard, *h_0_(t)*, and the exponential function of the linear combination of the predictors. Thus, the predictors have a multiplicative or proportional effect on the predicted hazard.

The partial likelihood function above, applied to estimate coefficients (*β*) is based on the assumption that only one seed germinates at a specific point in time. In our case, there were several germinating seeds at a specific time, so the problem of “ties” should be dealt with. In order to address the issue, the statistical software used in this work applied the Efron approximation to the exact likelihood [48].

The two different treatments (control and −0.6 MPa) and genotypes were also compared with respect to their hazards using the hazard ratio from the data organized to conduct the log-rank test. This parameter is analogous to an odds ratio in the setting of multiple logistic regression analysis and it is the ratio of the total number of observed to expected events in two independent comparison groups. The ratio of the number of germination events observed to those expected assuming the null hypothesis, *O/E*, represents the relative germination hazard rate of a seeds lot compared to another:

*HR* = (*O_a_*/*E_a_*)/(*O_b_*/*E_b_*) (4)

In order to test the significance of the individual regression coefficients, the Wald test was performed. Having assumed the coefficient *β* as approximately normally distributed, and having calculated the standard error of *β*, a *z*-test to determine if the value of *β* differed significantly from 0 was performed. Squaring the *z*-values gives the Wald statistics, which is approximately distributed as a χ^2^.

The statistical software used in this work did not allow to calculate the so called “frailty effects” due to the random differences in germination among different replicates [49]. However, to reduce possible differences among Petri dishes, we used a turnover change of their position inside the climatic chamber.

### 3.5. Parametric Approach

In addition to non-parametric and semi-parametric models, a parametric survival model was applied in the present study. In this case, a specific form for the survival distribution was assumed. Namely, the Accelerated Failure Time (AFT) model was adopted. The model postulates a direct relationship between the predictors and the survival time [36].

Let *S_0_*(*t*) represent the baseline germination function for the reference group, that is the control sample of the genotype Sh, and *S*(*t*) the germination function for the stressed seeds and the genotype Hy:

*S*(*t*) = *S*_0_(*γt*) (5)

where *γ* > 0 is a constant named “acceleration factor” that permits to evaluate the effect of predictor variables on the survival time and tells us how a change in covariate values changes the time scale from the baseline time scale [34] (p. 394).

The AFT assumption can also be expressed in terms of random variables for survival time:

*γt_0_* = *t* (6)

where *t_0_* is the baseline survival time for the combination genotype Sh/control and *t* is the analogous for stressed seeds and considering the genotype Hy. Gamma is a constant and represents the “time ratio”.

In our study, we considered an AFT model with two predictor variables: genotype (*X_1_*) and treatment (*X_2_*); the model can be expressed on the log scale as:

log(*t*) = *α_0_* + *α_1_X_1_* + *α_2_X_2_* + *ε* (7)

where *α_0_* is the logarithm of *t_0_*, the regression coefficients *α_1_* and *α_2_* represent the logarithm of the time ratios for factors *X_1_* and *X_2_* respectively. The term *ε* is a random error whose distribution depends on the *S(t)* distribution.

In order to find the most suitable distribution and assess the potential for an AFT model, four parametric distributions (Weibull, Log-normal, Log-logistic and Exponential) were fitted and compared. Adequacy of the AFT models for the data was initially evaluated by plotting the log of time against a linear function of the cumulative hazard rate. Although distributions with multiple parameters defining their shape may have a better fit, we also relied on a penalized metric provided by model selection indices such as Akaike Information Criterion (AIC) [50].

## 4. Conclusions

Time-to-event analysis proved to be a reliable tool to analyze germination data. However, the choice of the most appropriate method will depend on the purpose for which the analysis has to be run. The Kaplan-Meier (KM) survival curves provide a useful first insight into the shape of the survival function for each treatment/genotype, focusing on not occurring germination event. Its results are graphically intuitive, and it could be a useful method to compare more groups of seeds through the log-rank family tests and in terms of median or quartile of survival times. At the same time, the non-parametric hazard function, providing an insight into the conditional failure rates, could be a more graphically intuitive tool for analyzing germination data because it focused on the event occurring. Moreover, applying this method, no parametric assumptions about germination time distributions are necessary.

The Cox’s PH model provides useful information when one needs to consider a set of covariates that influence germination simultaneously. However, some constraints about results interpretation, such as the delay in the onset of germination, should be taken into account to assess the validity of the model when applied to germination data [17], and the PH assumptions need to be verified before applying this method. If the assumptions are met, the model could be considered as a useful tool for studies about the germination potential in seeds sown in peculiar environments or to test different seed storage conditions, adjusting parameters such as temperature and relative humidity to estimate which factor is more important in determining the germination decay over time.

Finally, the advantage of the accelerated failure time approach is that the effect of covariates on survival can be described in absolute terms rather than relative terms as a hazard ratio. Furthermore, being the shape of germination baseline hazard usually known, there is a good chance to use the suitable distribution form.

The AFT model can be interpreted in terms of the speed of those physiological processes that end with the germination event, and for this reason, it could be effective in those experiments whose aim is to evaluate the speed of germination in response to environmental factors such as testing the effects of an herbicide on germination of certain weeds or evaluating the germination precocity of some varieties compared to others in standard conditions or after specific treatments.

## Figures and Tables

**Table 1:** Results of the comparison tests for survival functions between stress treatments within each genotype (left side of the table) and between genotypes for each treatment (right side of the table). The asterisk on *p*-values indicates a statistical significance (*α* < 0.05). *z*-values are standardized. G stands for genotype, T for treatment.

**G**	**0 MPa vs. −0.6 MPa**					**T**	**Sh vs. Hy**			
**Sh**	Test	χ^2^	df	*p*-value	*z*-value	**No-Stress**	χ^2^	df	*p*-value	*z*-value
	M-H Log-rank	50.012	1	<0.0001	±7.072		4.432	1	0.0353 *	±2.105
	Gehan-Wilcoxon	38.361	1	<0.0001	±6.194		2.588	1	0.1077	±1.609
	Tarone-Ware	43.982	1	<0.0001	±6.632		3.359	1	0.0668	±1.833
	Peto-Peto	38.428	1	<0.0001	±6.199		2.354	1	0.1249	±1.534
	Modified Peto-Peto	38.380	1	<0.0001	±6.195		2.348	1	0.1254	±1.532
	Fleming-Harrington	48.561	1	<0.0001	±6.969		5.602	1	0.0179 *	±2.367
**Hy**	M-H Log-rank	43.580	1	<0.0001	±6.602	**Stress**	2.972	1	0.0847	±1.724
	Gehan-Wilcoxon	36.546	1	<0.0001	±6.045		2.588	1	0.1077	±1.609
	Tarone-Ware	40.225	1	<0.0001	±6.342		2.799	1	0.0943	±1.673
	Peto-Peto	36.321	1	<0.0001	±6.027		2.452	1	0.1174	±1.566
	Modified Peto-Peto	36.292	1	<0.0001	±6.024		2.451	1	0.1175	±1.565
	Fleming-Harrington	39.032	1	<0.0001	±6.248		2.741	1	0.0978	±1.656

**Table 2:** Summary table of the Cox’s PH model for germination data. The *z-*value of the Wald test tests the hypothesis that *βi* = 0 against the alternative *βi* ≠ 0. *p*-value represents the probability of obtaining a *z*-value larger in absolute value than the one obtained. The asterisk on *p*-values indicates a statistical significance (*α* < 0.05).

Explanatory Variable	*β_i_*	SE of *β_i_*	Exp (*β_i_*)	Wald *z*-Value	*p*-Value	Exp (*β_i_*) 95% CI
Genotype (Hy)	−0.2344	0.087	0.791	−2.6875	0.0072 *	0.6667–0.9385
Treatment (stress)	−0.8685	0.090	0.419	−9.6221	<0.00001	0.3515–0.5008

**Table 3:** Shape (s) and scale (m) parameters of the log-normal and log-logistic distributions as estimated by the Maximum Likelihood Estimates (MLE). SE represents the standard error.

**Log-Normal Distribution**						
**Genotype**	**Treatment (ψ in MPa)**	**Shape (s)**	**SE**	**Scale (m)**	**SE**	**−2 Log-Likelihood**
**Sh**	0	1.311	0.038	0.538	0.029	747.08
	−0.6	1.550	0.092	1.109	0.084	618.34
**Hy**	0	1.425	0.044	0.601	0.036	740.10
	−0.6	1.835	0.114	1.242	0.105	565.32
**Log-Logistic Distribution**						
**Sh**	0	1.315	0.037	0.304	0.019	742.94
	−0.6	1.497	0.095	0.709	0.058	620.46
**Hy**	0	1.394	0.043	0.355	0.024	738.74
	−0.6	1.792	0.117	0.800	0.074	567.14

**Table 4:** Summary table of the AFT model (log-normal and log-logistic distribution). *z*-values test the hypothesis that the parameter value is zero. *p*-values test the significance of the corresponding parameter. The asterisks on *p*-values indicate a statistical significance (α < 0.05).

**Log-Normal Model**							
**Parameter**	**Parameter Estimate**	**SE**	***z*-Value**	***p*-Value**	**95% CI**	**T50**	**Time Ratio (γ)**
Intercept	1.3185 (α_0_)	0.0403	32.68	<0.0001	1.24–1.40	3.7381	1
Treatment	0.4520 (α_1_)	0.0482	9.36	<0.0001	0.35–0.54	5.8747	1.5715
Genotype	0.1151 (α_2_)	0.0481	2.39	0.0167 *	0.02–0.20	4.1944	1.1220
**Log-Logistic Model**							
**Parameter**	**Parameter Estimate**	**SE**	***z*-value**	***p*-value**	**95% CI**	**T50**	**Time Ratio (γ)**
Intercept	1.3086 (α_0_)	0.0397	32.92	<0.0001	1.23–1.38	3.7013	1
Treatment	0.4554 (α_1_)	0.0487	9.34	<0.0001	0.36–0.55	5.8364	1.5768
Genotype	0.1184 (α_2_)	0.0493	2.40	0.0163*	0.02–0.21	4.1666	1.1257

## Abbreviations

AFT	Accelerated Failure Time
F-H	Fleming- Harrington
KM	Kaplan Meier
M-H	Mantel-Haenszel
PH	Proportional Hazard
